# Invasive Colonic Entamoebiasis in Wild Cane Toads, Australia

**DOI:** 10.3201/eid2408.180101

**Published:** 2018-08

**Authors:** Cathy M. Shilton, Jan Šlapeta, Richard Shine, Gregory P. Brown

**Affiliations:** Northern Territory Department of Primary Industry and Resources, Darwin, Northern Territory, Australia (C.M. Shilton);; University of Sydney, Sydney, New South Wales, Australia (J. Šlapeta, R. Shine, G.P. Brown)

**Keywords:** amphibian, wildlife, Bufo marinus, invasive species, Australia, ameba, parasites, entamoebiasis, cane toads, Rhinella marina

## Abstract

We detected a disease syndrome in free-ranging Australian cane toads involving atypical behavior and emaciation that is associated with a previously undescribed *Entamoeba* sp. that infiltrates the colonic lining, causing it to slough. The organism may become seasonally pathogenic when toads are under hydric and nutritional stress.

The emergence of new diseases in wildlife substantially threatens global biodiversity in many taxa (*1*), but amphibians face unusually high risk for pathogen-mediated population declines (*2*,*3*). Disease outbreaks among invasive amphibians are of particular concern because the invader may imperil native fauna by transmitting new pathogens (*1*). We documented severe (lethal) colitis of wild cane toads (*Rhinella marina*) in Australia associated with *Entamoeba* spp.

Cane toads were introduced to eastern Australia in 1935 and have now spread 2,000 km westward across the continent. The disease outbreak was observed at the University of Sydney Tropical Ecology Research Facility (TERF), in Australia’s Northern Territory. The area experiences a wet–dry tropical climate, with high temperatures year-round but with rainfall limited to a 6-month wet season (November–May). Cane toads reached TERF in 2005, and the disease outbreak occurred 9 years later.

## The Study

In August 2014, we noticed dead and moribund toads around the grounds of TERF. In daylight, emaciated toads were found sitting in puddles of water formed under the building’s air conditioners. These diurnal observations were unprecedented; toads at this site were normally nocturnal and seen hydrating only in this manner at night. In addition, on several mornings, we observed moribund toads on open areas of lawn, fully exposed to sunlight and apparently too weak to seek refuge. During September and October 2014, we euthanized and necropsied 22 toads found hydrating or otherwise diurnally active near the TERF buildings. For comparative purposes we also necropsied 2 other groups of toads: 7 collected during November 2014 from a lagoon 30 km from TERF and 8 collected during February 2015 from the TERF grounds (Table 1).

**Table 1:** Summary of morphologic, pathologic, and prevalence data from cane toads (*Rhinella marina*) sampled for amebiasis, tropical Australia*

Collection site, latitude, longitude	Collection date (season)	No. collected	Body length, mm, mean ±SE	Body mass, g, mean ±SE	Body condition, mean ±SE†	Illness score mean ±SE	No. with invasive amebiasis	Colonic lesion severity, mean ±SE‡	No. sequenced	No. OTU_12 positive	No. *Entamoeba ranarum* positive
TERF, 12.579°S, 131.314°E	2014 Sep–Oct (dry)	22	89.2 (± 3.3)	70.4 (± 9.1)	−0.07 (± 0.08)	2.2 (± 0.2)	21	2.0 (± 0.3)	5	5	0
	2015 Feb (wet)	8	93.5 (± 5.2)	114.3 (± 24.4)	0.27 (± 0.07)	0.3 (± 0.2)	3	−0.4 (± 0.7)	8	7	2
Lagoon, 12.714°S, 131.419°E	2014 Nov (dry)	7	88.4 (± 7.7)	71.3 (± 21.9)	−0.09 (± 0.08)	0.3 (± 0.3)	1	−1.4 (± 0.1)	5	5	3

*OTU, operational taxonomic unit; TERF, University of Sydney’Tropical Ecology Research Facility (New South Wales, Australia). †Body condition scores are residuals calculated from regression of ln-transformed body mass on ln-transformed body length. ‡Colonic lesion severity is a conglomerate statistical measure (principal component) of 4 scores of lesion severity. Higher values indicate increased severity.

**Figure 1:**
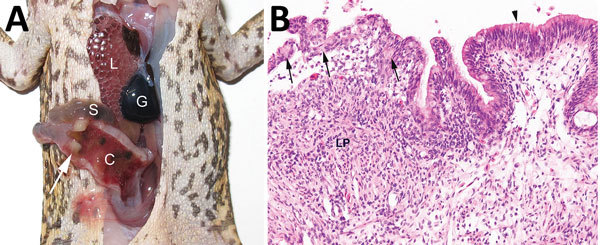
Invasive colonic entamoebiasis in wild cane toads (*Rhinella marina*), tropical Australia, 2014–2015. A) Toad with severe colonic amebiasis. The colon (C) has been opened to show intraluminal hemorrhagic content and blood clots. There is segmental full-thickness necrosis of the colon wall (white arrow). Lung (L), small intestine (S), and gall bladder (G) are annotated for perspective. B) Photomicrograph of colonic amebiasis. The affected segment of mucosal epithelium, which contains several amebae (arrows) is jumbled and sloughing from the underlying lamina propria (LP). Relatively normal colonic epithelium is present at right (arrowhead). There is lymphohistiocytic and granulocytic infiltration of the lamina propria underlying the affected epithelium. Hematoxylin and eosin stain. Original magnification ×200.

We detected invasive amebiasis by histologic analysis in all 3 groups, but disease was most prevalent and intense in the dry-season TERF toads (Table 1; Technical Appendix). The most severe cases were detected in toads in poor body condition with overt illness (online Technical Appendix). Gross pathologic findings ranged from no obvious lesions in mildly affected toads to thickened colonic walls with hyperemic serosal vasculature and hemorrhagic content in severely affected toads (Figure 1, panel A). Histologically appreciable lesions (invasive amebiasis) were commonly limited to the colon, although in severely affected toads, lesions extended through the small intestine and, rarely, into the stomach. The intestinal mucosal epithelium was variably hyperplastic, showing moderate to marked lymphoplasmacytic infiltration, to eroded or deeply ulcerated, showing associated granulocyte and macrophage infiltration. Organisms consistent in morphology with *Entamoeba* spp. were among mucosal epithelial cells, often near the basement membrane and rarely within the lamina propria (Figure 1, panel B; Technical Appendix) and not present in other organs.

**Figure 2:**
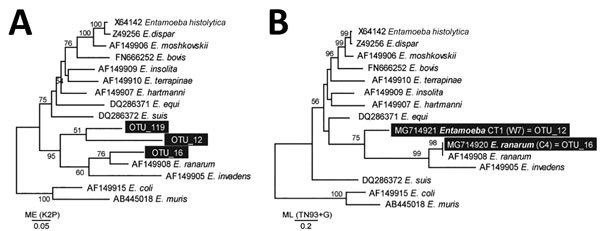
Phylogenetic inference of cane toad (*Rhinella marina*) *Entamoeba* SSU-rDNA sequences. *Entamoeba* SSU-rDNA sequences obtained using environmental next-generation amplicon sequencing (A) and conventional amplification using *Entamoeba*-specific primers (B) were aligned with available representative SSU-rDNA sequences. Each sequence is accompanied by GenBank accession number and *Entamoeba* species name. New sequences are in black boxes. Bootstrap support values (500 replicates) are shown next to the branches. The evolutionary distances were computed using the maximum-likelihood method and are in the units of number of base substitutions per site (SCALE BARS). New sequences are representative of the OTU contigs (A) or are sequences directly from PCR amplicon (B). OTU, operational taxonomic unit; SSU, small subunit.

We applied environmental DNA sequencing to identify the community of eukaryotes (diversity profile) within the colons of 8 infected and 10 uninfected animals based on histopathologic investigation. From the 18 colon scrapings, we obtained 1,365,109 eukaryotic V1–V3 small subunit (SSU)–rDNA high-quality Illumina MiSeq (Illumina, San Diego, CA, USA) reads clustered into operational taxonomic units (OTU). Three OTUs demonstrated perfect or high-percentage identity with SSU rDNA sequences of the amebae in the genus *Entamoeba*: *E. ranarum* (OTU_16) and 2 new cryptic species (OTU_12 and OTU_119 [Figure 2]). Using SSU-rDNA *Entamoeba* species–specific primers, we confirmed the presence of *E. ranarum* (OTU_16) and *Entamoeba* sp. CT1 (OTU_12) (GenBank accession nos. MG714920–MG714921). The new *Entamoeba* sp. CT1 (OTU_12) was significantly more abundant in toads with histologically diagnosed invasive amoebiasis (t = 2.2, d.f. = 16, p = 0.04; Table 2) and significantly more abundant in toads with more severe colonic lesions (F_1,16_ = 7.0, p = 0.017). OTU_12 was also detected at low levels in clinically healthy toads without histologic evidence of invasive disease from the site 30 km away from TERF (Table 1). *Entamoeba ranarum* (OTU_16) was no more prevalent or abundant in diseased toads than in healthy conspecifics, suggesting that OTU_12 (rather than *E. ranarum*) is the causative agent of the colitis.

**Table 2:** Ameba abundance from the colons of wild cane toads (*Rhinella marina*), tropical Australia

OTU no.	Ameba species	OTU abundance, %	

Diseased, animal no.									Healthy, animal no.										p value
W7	W11	W14	W21	W22	W28	W29	W30	C9	C10	C11	C12	C1	C2	C3	C4	C7	C8
OTU_12	*Entamoeba* sp. CT1	26.68	2.33	1.07	0.20	41.47	0.42	54.96	0.42		0.89	0.59	0.71	0.64	0.72	0.58	0.30	0.24	0.22	0.00	**0.04**
OTU_16	*Entamoeba ranarum*	0.00	0.00	0.00	0.00	0.00	0.21	0.00	3.76		0.00	0.00	0.00	0.00	1.20	0.00	35.89	13.64	0.00	0.00	0.29
OTU_119	*Entamoeba* sp.	0.00	0.00	0.00	0.00	0.00	0.00	0.00	0.00		0.00	0.00	0.00	2.14	0.00	0.00	0.00	0.00	0.00	0.00	0.39
OTU_25	*Naegleria* sp	0.00	0.00	23.07	1.02	0.68	0.00	0.00	0.00		0.22	0.00	0.24	0.00	0.24	0.00	0.00	0.00	0.22	0.00	0.25
OTU_106	*Acanthamoeba* sp	0.00	0.00	0.00	0.00	0.00	0.00	0.00	0.00		0.00	0.00	0.00	2.78	0.24	0.00	0.00	0.00	0.00	0.00	0.35
OTU_132	*Hartmannella vermiformis*	0.00	0.00	0.00	0.00	0.00	0.00	0.00	0.00		0.22	0.00	0.00	1.28	0.00	0.00	0.00	0.00	0.00	0.23	0.24

*p values refer to differences in abundance between diseased and healthy animals, determined by *t* tests. Bold type indicates significance (p<0.05).

Although biologists had monitored toads at the site since 2005, no unusual mortality was observed until 2014. The disease outbreak involved conspicuous behavior, severe clinical disease, and high mortality. Populations of invasive species (including Australian cane toads) often collapse after establishment, but the causes usually are unclear (*4*). An investigation into declines of Australian cane toad populations (*5*) posited an unknown microbial disease as a possible cause. Plausibly, OTU_12 could be that unknown pathogen. It might have remained undetected until now because rapid postmortem decomposition of the colon lining obscures lesions. Euthanizing toads in the final stages of the disease and immediately fixing their tissue enabled us to detect the lesions histologically.

## Conclusions

To our knowledge, the only published description of pathology associated with amebic infection in amphibians is a case of renal disease in a single captive cane toad (*6*). Although a recent survey of cane toads in Puerto Rico recorded 2 animals with histologic evidence of amebic enteritis (*7*), extensive surveys of intestinal protozoa in Australian toads did not detect amebiasis (*8*). In other wild anurans, amebas (including *Entamoeba* spp.) sometimes are evident cytologically in the intestine (*9*) but have never been linked to disease.

The genus *Entamoeba* infects a range of taxa, often as commensals, and less commonly as pathogens (*10*,*11*). In humans, *E. histolytica* is associated with extensive illness and death (*12*,*13*). However, the presence of *Entamoeba* is inconsistently associated with disease and might depend on interactions between the environment, host, and parasite (*12*,*13*). For example, poor nutritional status facilitates invasive amebiasis in humans (*12*–*14*). Likewise, anorexia predisposes captive herpetofauna to invasive entamoebiasis (*11*). Furthermore, interactions between *Entamoeba* spp. and other organisms in the gut microbiome may affect growth or virulence of the pathogen (*11*,*12*).

Based on this pattern of *Entamoeba* pathogenesis in other species and on knowledge of toad ecology, we speculate the following scenario for the disease outbreak. Toads ingest encysted OTU_12 by foraging on the ground where an infected host has defecated (*12*). Rates of infection increase during the dry season when toads congregate nightly around dwindling water sources (*5*,*15*). Dry-season congregations of toads also decrease food intake as competition for food increases (*15*). Decreased feeding alters the intestinal microbiome and causes *Entamoeba* in the colon to activate genes that enables it to feed on epithelial cells instead of colon contents. Destruction of the colon wall causes fluid imbalance, forcing toads to remain in moist areas to prevent dehydration. As destruction of the colon wall progresses, bacterial infection leads to septicemia, anorexia, and eventual death. Further experimental studies are needed to verify this conjectured chain of causation.

The circumstances underlying the unprecedented mortality event and its implications require further investigation. Of paramount importance is determining the current distribution of OTU_12, its original host, and whether native frog populations are at risk from the disease. Isolating and culturing OTU_12 for reference material and morphologic characterization of cysts and trophozoites would facilitate further study. Determining whether changes in the environment, microbiome, or both cause *Entamoeba* to switch from commensal to pathogenic and the role the disease may play in controlling populations of cane toads also warrant further study.

Technical Appendix Additional methods and results for study of invasive colonic entamoebiasis in wild cane toads, Australia.
