# Supplementary material for: Invasive Colonic Entamoebiasis in Wild Cane Toads, Australia
Source: Emerg Infect Dis. 2018 Aug;24(8):1541–3. doi: 10.3201/eid2408.180101 (PMC6056091; doi:10.3201/eid2408.180101)
Supplement: Technical Appendix — Additional methods and results for study of invasive colonic entamoebiasis in wild cane toads, Australia. [file 18-0101-Techapp-s1.pdf]

Article DOI: https://doi.org/10.3201/eid2408.180101

Invasive Colonic Entamoebiasis in Wild Cane Toads, Australia

Technical Appendix

Sample Collection and Histology

During October 2014–February 2015, we collected 37 toads from the vicinity of the University of Sydney's Tropical Research Facility (TERF) and a lagoon 30 km away (Table 1; Technical Appendix Figure 1). We subjectively assessed 26 of the 37 toads for signs of clinical illness immediately after collection. Illness was scored as follows: 0 (normal), 1 mild (slightly sluggish movement), 2 moderate (more sluggish, and slightly uncoordinated movement), 3 marked (flaccid when handled and reduced or absent righting ability). The body condition of each toad was calculated using residuals from a linear regression of ln-transformed body mass on ln-transformed body length.

Toads were euthanized by intracoelomic injection of sodium pentabarbital and the colon and contents removed for histologic examination and eukaryotic diversity profiling. All methodology was approved by the University of Sydney's Animal Ethics Committee (permit # 2013/5989). For histology, colon tissue was fixed in 10% buffered formalin and processed in standard fashion. Sections were cut at 5 μm and stained with hematoxylin and eosin or periodic acid-Schiff. In addition, colonic impression smears were made for a subsample (n = 16) of infected and uninfected toads and stained with Wright’s stain.

Toads were classified as having invasive amoebiasis if amoebas were histologically visible within the intestinal mucosa (e.g., Technical Appendix Figure 2). In addition to designating amoebiasis as being present or absent in each toad, we also scored the severity of colonic lesions as follows:

i. Type of lesion observed in the mucosal epithelia: scored as 0 (normal), 1 (lesion limited to epithelial hyperplasia, attenuation or single cell necrosis), 2 (multifocal erosion or ulceration present), or 3 (extensive erosion or ulceration present).

ii. Severity of lesion in the mucosal epithelia: scored as 0 (no lesion), 1 (mild), 2 (moderate), or 3 (severe).

iii. Degree of inflammation in the wall of colon: scored as 0 (no inflammation) 1 (mild), 2 (moderate), or 3 (severe).

iv. Number of amoebas observed within the intestinal mucosa: scored as 0 (none), 1 (rare to occasional), 2 (moderate numbers), or 3 (abundant).

Statistical Analyses of Clinical and Histologic Data

These 4 scalar variables describing the colonic lesions of each toad were amalgamated into a single score using principal component analysis. We used the first component (PC1) produced from this analysis as an overall measure of the severity of colonic amoebiasis.

Abundances of each of the 6 main protist taxa identified from toad colon scrapings were ln-transformed and regressed against lesion severity to assess which organisms were most closely associated with the observed colonic pathology. All statistical tests were carried out using JMP11 (SAS Institute, Cary NC, USA).

Results

Toads in the TERF dry-season sample had higher prevalence (N = 37, χ^2 = 22.1, p<01; Technical Appendix Figure 4) and severity ($F_{1,34}$ = 14.1, p<0.0001; Technical Appendix Figure 4) of colonic amoebiasis (PC1) than toads in the other 2 collection groups (Technical Appendix Figure 4). Toads with more severe amoebiaisis were in poorer body condition ($F_{1,35}$ = 10.5, p = 0.0027; Technical Appendix Figure 5) and had more overt signs of clinical illness ($F_{1,24}$ = 29.2, p<0.0001; Technical Appendix Figure 5).

Among the 18 toads for which colon linings were sampled for protists using next-generation sequencing (NGS), only the abundance of operational taxonomic unit (OTU) _12 was significantly correlated with the severity of colonic lesions ($F_{1,16}$ = 7.0, p = 0.017; Technical Appendix Figure 6). Abundances of other the amoeboids were not related to lesion severity (all $F_{1,16}$<2.3, all p>0.15). Although the abundance of OTU_12 was correlated with lesion severity, it was not related to clinical illness score or to body condition (both F<2.8, both p>0.12). Thus, since some clinically healthy, fat toads had relatively high abundances of

OTU_12, the presence of the organism alone was not a reliable indicator of illness. In addition, OTU_12 was equally abundant among toads from the 3 sample groups ($F_{2,15} = 2.1$, $p = 0.16$).

Molecular Identification and Next-Generation Sequencing Eukaryotic Diversity Profiling

DNA was isolated from 18 colon scrapings from a selection of diseased and apparently healthy cane toads (*Rhinella marina*) (Table 2). Total DNA was isolated from 250 mg of feces using the PowerSoil Kit (Mo-Bio Laboratories, Carlsbad, CA, USA) with a FastPrep-24 Instrument and setting 6 for 40 s (MP Biochemicals, Santa Ana, CA, USA). Extracted DNA was eluted into 100 µL of ddH$_2$0 water and stored at −20°C. All DNA was isolated together with negative controls in each batch. DNA samples were shipped to the MR DNA (http://www.mrdnalab.com, Shallowater, TX, USA) for PCR and MiSeq Illumina (Illumina, San Diego, CA, USA) sequencing.

The 18S rRNA gene (small subunit [SSU]–rDNA) diversity profiling assay was based on the primer pairs (i) Euk7A (AAC CTG GTT GAT CCT GCC AGT) and Euk570R (GCT ATTGGA GCT GGA ATT AC), amplifying ≈500–550 bp of the 5′-end of the eukaryotic SSU-rDNA (hypervariable V1–V3 region). Amplification and Illumina sequencing was performed at MR DNA. The bar-coded PCR amplicons were pooled and sequenced on the Illumina MiSeq platform, using Illumina TruSeq DNA library Single End sequencing chemistry. Sequence data were processed using MR DNA analysis pipeline. Raw sequence libraries were trimmed to a minimum length of 150 bp, depleted of bar codes, and subjected to a filtering step using the quality scores file to remove sequences with any anomalous base calls. Unique sequences were aligned using in-house curated database specific for SSU-rDNA. The resulting unique sequences were preclustered to remove amplification and sequencing errors and chimeras were identified and removed using UCHIME (*1*). OTUs were defined by clustering at 3% divergence (97% similarity). Final OTUs were taxonomically classified using BLASTn against a curated database derived from the National Center for Biotechnology Information (http://www.ncbi.nlm.nih.gov). OTUs taxonomically classified as amoeba were further verified by recompiling the reads within each OTU to manually check for any potentially cryptic OUT that would be masked by 3% threshold; such analysis did not reveal any cryptic OTU.

Partial SSU-rDNA was amplified using primers ENTAGEN_F [S0517] (ACT TCA GGG GGA GTA TGG TCAC) and ENTAGEN_R [S0518] (CAA GAT GTC TAA GGG CAT CAC AG) (*2*). Reactions of 30 μL contained MyTaq Red Mix (BioLine, Alexandria, NSW, Australia) and ≈10–50 ng of genomic DNA template (2 μL). The cycling conditions were as follows: denaturing at 95°C for 1 min followed by 35 cycles of 95°C for 15 s, 60°C for 15 s, 72°C for 30 s, and a final elongation for 5 min at 72°C. The PCRs were conducted in a Veriti Thermal Cycler (Applied Biosystems, Foster City, CA, USA) alongside sterile PCR-grade water as negative control. Resulting products were resolved in 1.5% (w/v) agarose. PCR products with single band product of the expected size were directly and bi-directionally sequenced after purification using amplification primers at Macrogen Inc. (Seoul, South Korea). Sequences were assembled, aligned with related sequences, and analyzed using CLC Main Workbench 6.9.1 (CLC bio, Aarhus, Denmark) and deposited in GenBank under accession nos. MG714920–MG714921.

Evolutionary analyses were conducted in MEGA7.0.14 (*1*). Multiple sequence alignment was constructed with all available SSU-rDNA sequences belonging to defined clades. Final alignment and analysis included selection of the main Entamoeba clades (*3*). For *Entamoeba* SSU-rDNA obtained using conventional PCR, nucleotide divergences were calculated using the maximum likelihood best fit model selected (TN93+G) using MEGA. Phylogenetic tree was inferred using maximum likelihood and the bootstrap support inferred from 500 replicates. For OTUs belonging to *Entamoeba* SSU-rDNA obtained using NGS, nucleotide divergences were calculated with the K2P method using MEGA. The phylogenetic tree was inferred using minimum evolution and the bootstrap support inferred from 1,000 replicates.

All raw/processed Illumina reads, OTU and alignments are available at LabArchives (http://dx.doi.org/10.6070/H4SN07G9).

Morphology of Presumptive Entamoeba OTU_12

We observed trophozoites and cysts on histologic sections and colonic impression smears from toads infected with OTU_12 (Technical Appendix Figures 2, 3). However, using morphologic features to distinguish among species of *Entamoeba* is often not possible. For instance, Entamoeba OTU_12 is genetically closely related to *E. ranarum* of amphibians and *E. invadens* of reptiles. The latter 2 species are morphologically grouped with other

Entamoeba sp. (including *E. histolytica*) by virtue of forming mature cysts that typically contain 4 nuclei (*4*,*5*) although on occasion, *E. histolytica* and *E. ranarum* may have up to 8 or 16 nuclei, respectively (*6*). The cytomorphology of all life stages of *E. ranarum* (including cysts) has been described as indistinguishable from *E. histolytica* (*7*,*8*). Likewise, the cytomorphology of *E. histolytica* and *E. invadens* has been described as identical (*9*). It seems likely, therefore, that *Entamoeba* OTU_12 will be morphologically very similar to *E. ranarum*, *E. invadens*, and *E. histolytica* in both the trophozoite and cyst stages. Based on observations of histologic sections and impression smears taken from colons of naturally infected cane toads in our study, this seems to be the case. The trophozoites (Technical Appendix Figure 2) and cysts (Technical Appendix Figure 3) we observed were morphologically consistent with these other *Entamoeba* species (*4*,*6*,*9*). We found cysts to be rare in cytological preparations of colon smears, present in only 3 of 8 smears from infected individuals. This scarcity of cysts has also been noted in frogs (*4*).

In most cases, we cannot be confident that the organisms we observed were actually OTU_12, because many toads were co-infected with other amoebae Table 2). Ideally, future studies can use axenic cultures (confirmed through NGS) to fully characterize the morphology of trophozoites and cysts of *Entamoeba* OTU_12. Techniques for establishing and maintaining axenic cultures of *Entamoeba* are well described in the literature, as are the use of preferred stains, such as iodine and iron hematoxylin, to characterize the morphology of different life cycle stages (*9*–*13*).

References

1. Edgar RC, Haas BJ, Clemente JC, Quince C, Knight R. UCHIME improves sensitivity and speed of chimera detection. Bioinformatics. 2011;27:2194–200. PubMed http://dx.doi.org/10.1093/bioinformatics/btr381

2. Kumar S, Stecher G, Tamura K. MEGA7: Molecular Evolutionary Genetics Analysis version 7.0 for bigger datasets. Mol Biol Evol. 2016;33:1870–4. PubMed http://dx.doi.org/10.1093/molbev/msw054

3. Stensvold CR, Lebbad M, Victory EL, Verweij JJ, Tannich E, Alfellani M, et al. Increased sampling reveals novel lineages of E*ntamoeba*: consequences of genetic diversity and host specificity for taxonomy and molecular detection. Protist. 2011;162:525–41. Erratum in: Protist. 2016;167:31. PubMed http://dx.doi.org/10.1016/j.protis.2010.11.002

4. Dobell CC. Researches on the intestinal protozoa of frogs and toads. J Cell Sci. 1909;2:201–78.

5. Hooshyar H, Rostamkhani P, Rezaeian M. An annotated checklist of the human and animal entamoeba (Amoebida: Endamoebidae) species—a review article. Iran J Parasitol. 2015;10:146–56. PubMed

6. Noble ER, Noble GA. Parasitology: the biology of animal parasites. 6th ed. Philadelphia: Lea & Febiger; 1989.

7. Dobell C. Are *Entamoeba histolytica* and *Entamoeba ranarum* the same species? An experimental inquiry. Parasitol. 1918;10:294–310. http://dx.doi.org/10.1017/S0031182000003875

8. Taliaferro WH, Fisher AB. The morphology of motile and encysted *Endamoeba ranarum* in culture. Ann Trop Med Parasitol. 1926;20:89–96. http://dx.doi.org/10.1080/00034983.1926.11684480

9. Geiman QM, Ratcliffe HL. Morphology and life-cycle of an amoeba producing amoebiasis in reptiles. Parasitol. 1936;28:208–28. http://dx.doi.org/10.1017/S0031182000022423

10. Clark CG. Axenic cultivation of *Entamoeba dispar* Brumpt 1925, *Entamoeba insolita* Geiman and Wichterman 1937 and *Entamoeba ranarum* Grassi 1879. J Eukaryot Microbiol. 1995;42:590–3. PubMed http://dx.doi.org/10.1111/j.1550-7408.1995.tb05912.x

11. McConnachie EW. Studies on *Entamoeba invadens* Rodhain, 1934, in vitro, and its relationship to some other species of *Entamoeba*. Parasitology. 1955;45:452–81. PubMed http://dx.doi.org/10.1017/S0031182000027803

12. Robinson GL. The laboratory diagnosis of human parasitic amoebae. Trans R Soc Trop Med Hyg. 1968;62:285–94. PubMed http://dx.doi.org/10.1016/0035-9203(68)90170-3

13. González-Ruiz A, Haque R, Aguirre A, Castañón G, Hall A, Guhl F, et al. Value of microscopy in the diagnosis of dysentery associated with invasive *Entamoeba histolytica*. J Clin Pathol. 1994;47:236–9. PubMed http://dx.doi.org/10.1136/jcp.47.3.236

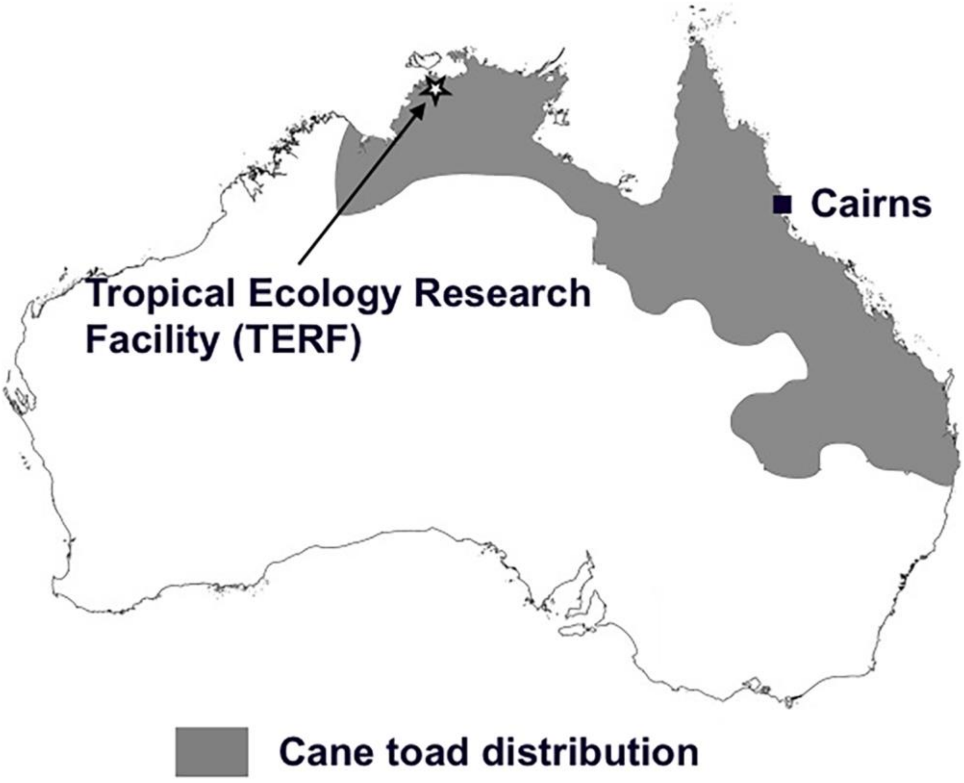

Technical Appendix Figure 1. Current approximate distribution of cane toads (*Rhinella marina*) in Australia and the location of the study area. Toads were introduced at Cairns in 1935 and have spread 2,000 km westward since then.

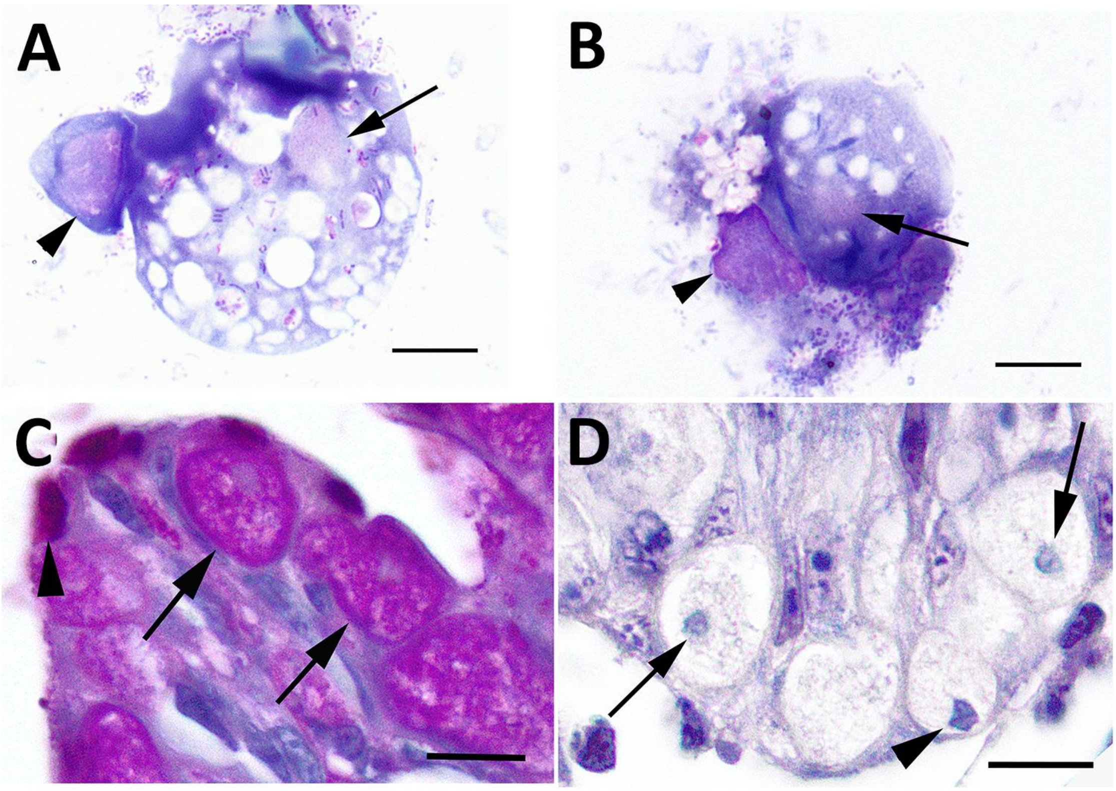

Technical Appendix Figure 2. Morphology of presumptive *Entamoeba* operational taxonomic unit (OTU) _12 trophozoites. A, B) Cytomorphology from colonic impression smears stained with Wright's stain, in a toad in which the only amoeba identified by next-generation sequencing was *Entamoeba* OTU_12. C, D) Histomorphologic findings from a toad with colonic amoebiasis in which the only *Entamoeba* spp. detected with NGS was OTU_12. A) Large trophozoite showing pale staining nucleus (arrow) and abundant vacuolated cytoplasm contained ingested bacteria. Note toad lymphocyte (arrowhead). B) Smaller precystic trophozoite with ill-defined pale-staining nucleus (arrow) and several elongate dark blue chromatoid bodies in cytoplasm. Note unidentifiable toad cell nucleus stripped of cytoplasm (arrowhead). C) Trophozoites within sloughing colon epithelium. Note small, indistinct pale blue nuclei visible in 2 trophozoites (arrows). Trophozoite cytoplasm contains abundant magenta-staining glycogen. Note mucus within toad intestinal mucosal goblet cells stains a darker shade of magenta (arrowhead). Periodic acid-Schiff stain. D) Trophozoite nuclei are small, with dark rim of heavily staining peripheral chromatin packed against the nuclear membrane and small, central karyosome (arrows). In 1 nucleus, "spoke radii" are appreciable, extending from the karyosome to peripheral chromatin (arrowhead). Phosphotungstic acid hematoxylin. All scale bars = 10 µm.

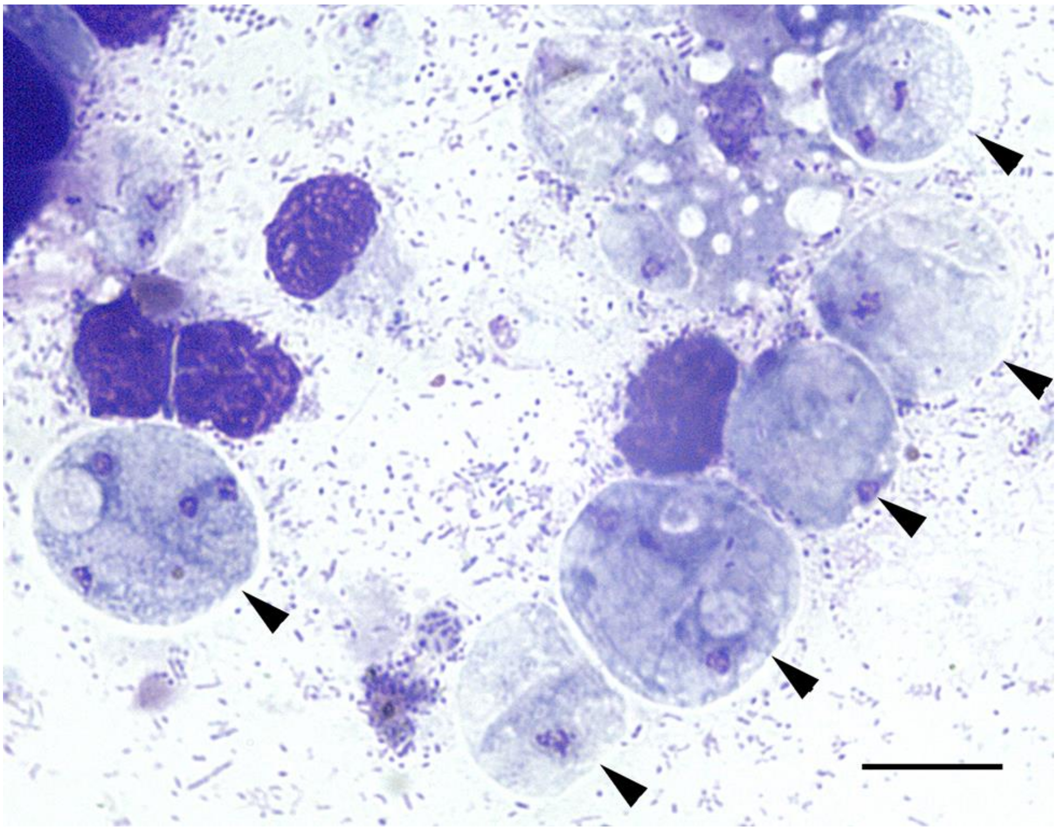

Technical Appendix Figure 3. Morphologic findings of presumptive *Entamoeba* operational taxonomic unit (OTU) _12 cysts from colonic impression smear stained with Wright's stain, in a toad in which the only *Entamoeba* sp. identified by next-generation sequencing was OTU_12. Cysts (arrowheads) typically contained 1–4 small nuclei, although some contained up to 8 nuclei. Note thin clear zone (wall) surrounding cysts and large, clear glycogen vacuoles visible in some cysts. Scale bar = 10 µm.

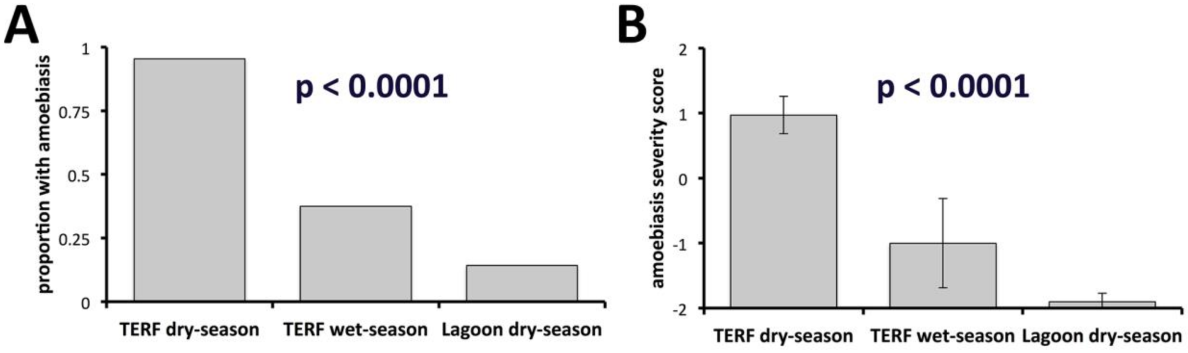

Technical Appendix Figure 4. Prevalence and severity of histologically diagnosed amoebiasis in three samples of cane toads (*Rhinella marina*). Although the disease was significantly most prevalent and severe at the time and place where the outbreak was first noticed (TERF dry-season), amoebiasis was also detected in toads at a later date (TERF wet-season) and 30 km further away (Lagoon dry-season). TERF, University of Sydney's Tropical Research Facility.

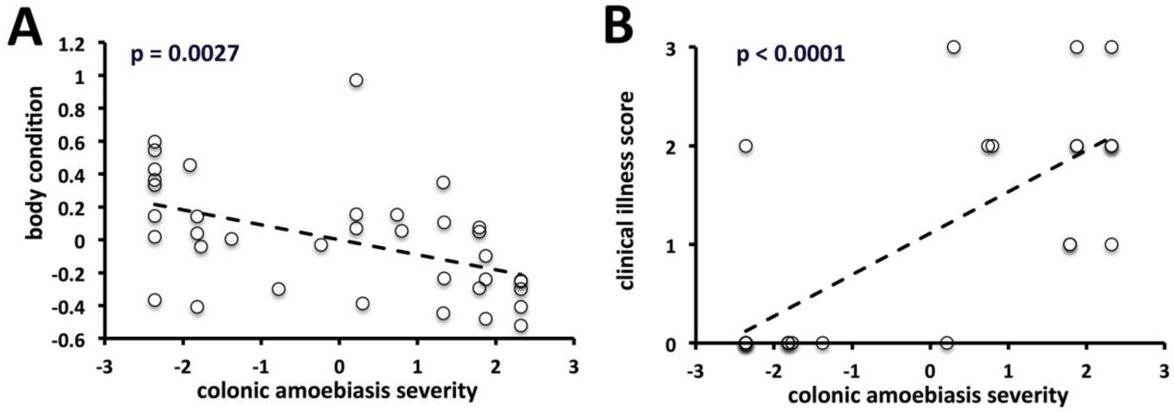

Technical Appendix Figure 5. Scatterplot of body condition (A) and clinical illness score (B) against histologic assessment of the severity of colonic lesions in cane toads (*Rhinella marina*). Toads with more severe lesions were significantly thinner and showed more serious signs of illness.

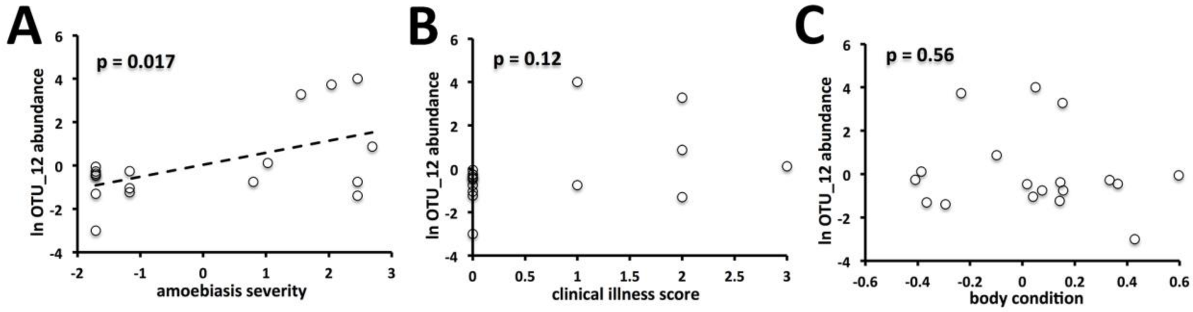

Technical Appendix Figure 6. Relationships between abundance of *Entamoeba sp.* CT1 (OTU_12) and severity of colonic amoebiasis based on histologic examination (A), level of clinical illness (B), and body condition in 18 cane toads (*Rhinella marina*) (C). OTU, operational taxonomic unit.
